# KRAS Mutation Detection by Real-Time and Digital PCR in Tumor Tissue and Plasma and Its Association with Survival in Stage II–IV Colorectal Cancer: A Kazakhstan Cohort Study

**DOI:** 10.3390/genes17080907

**Published:** 2026-07-31

**Authors:** Timur Nasrytdinov, Dilyara Kaidarova, Venera Israilova, Saken Khaidarov, Bayan Nurgaliyeva, Slu Izmailova, Gulmira Rapilbekova, Marat Rabandiyarov, Bibigul Abdygalyk, Askar Aidarov, Daulet Aidarov, Aray Aidarova

**Affiliations:** 1 Operating Theatre, Kazakh Institute of Oncology and Radiology, 91 Abay Ave., Almaty 050000, Kazakhstan; t.nasrytdinov@onco.kz; 2 Department of Oncology, Asfendiyarov Kazakh National Medical University, Tole-Bi Street 94, Almaty 050012, Kazakhstan; dilyara.kaidarova@gmail.com (D.K.); askar.a.e@mail.ru (A.A.); 3 Department of Cardiology, City Cardiology Centre, Faculty of Medicine, University of International Business, Abay Ave. 8A, Almaty 050010, Kazakhstan; israilova.venera@mail.ru; 4 Department of Molecular Biology and Medical Genetics, Asfendiyarov Kazakh National Medical University, Zheltoksan Street 37A, Almaty 050004, Kazakhstan; khaidarov.s@kaznmu.kz; 5 Department of Propaedeutics of Internal Medicine, Asfendiyarov Kazakh National Medical University, 94 Tole Bi Str., Almaty 050012, Kazakhstan; nurgalieva.b@kaznmu.kz; 6 Department of General Paediatrics, Children’s City Clinical Hospital No. 2 of Almaty, Microdistrict 2, Building 54, Almaty 050062, Kazakhstan; 7 Department of Gynaecology, University Medical Centre (UMC), Kerey and Zhanibek Khandar Str. 5/1, Astana 010000, Kazakhstan; gulmirarapilbek@gmail.com; 8 Department of Oncology, Kazakh-Russian Medical University, Abylay Khan Avenue 51/53, Almaty 050000, Kazakhstan; daulet_medik@mail.ru (D.A.); omar.al.askar20@gmail.com (A.A.)

**Keywords:** *KRAS*, colorectal cancer, digital PCR, liquid biopsy, circulating tumor DNA, real-time PCR, overall survival, molecular oncology, precision medicine, Kazakhstan

## Abstract

Background/Objectives: Colorectal cancer (CRC) is molecularly heterogeneous, and the *KRAS* (Kirsten rat sarcoma viral oncogene homolog) genotype both governs eligibility for anti-EGFR (epidermal growth factor receptor) therapy and carries prognostic weight. Central Asian data are scarce: no Kazakhstani series has described the *KRAS* variant spectrum, linked it to survival, or reported mutation detection rates across specimen types and PCR platforms. Methods: We studied 332 patients with morphologically confirmed stage II–IV colorectal adenocarcinoma. *KRAS* status was determined in formalin-fixed paraffin-embedded (FFPE) tumor tissue by allele-specific real-time PCR (RT-PCR) covering six selected codon 12 and 13 variants. Overall survival (OS) was estimated by the Kaplan–Meier method for the whole cohort and with stage stratification. Separately, mutation detection rates were recorded in three non-overlapping groups of different patients: plasma RT-PCR on the Idylla platform (*n* = 30), plasma nanoplate digital PCR (dPCR) on QIAcuity One (*n* = 120), and a routine tissue RT-PCR series (546 evaluable of 550). Because these groups differed in patients, specimen type, and mutation panel, this comparison describes observed detection rates only and supports no inference about analytical sensitivity, specificity, or concordance. Results: *KRAS* was mutated in 149/332 tumors (44.9%); codon 12 supplied 80.5% of variants, led by G12D (32.2%), G12V (24.8%) and G13D (19.5%). Median OS did not differ between mutant and wild-type tumors (39.0 vs. 36.6 months; *p* = 0.40). Variant-level differences were directionally consistent, but none was significant, and all were exploratory and unadjusted for multiplicity. Observed detection rates were 40.3% for tissue RT-PCR (220/546), 13.3% for plasma RT-PCR (4/30; continuity-corrected *p* = 0.006 vs. tissue), and 50.8% for plasma dPCR (61/120; continuity-corrected *p* = 0.044, Pearson *p* = 0.034 vs. tissue). *BRAF* V600E was detected by plasma dPCR in 11/120 cases (9.1%). Conclusions: This first Kazakhstani series places *KRAS* frequency within the internationally reported range and shows that variant-level reporting reveals prognostic structure that a binary call conceals. The higher detection rate seen with plasma dPCR is hypothesis-generating, not evidence of platform superiority, and motivates a prospective paired-sample study with harmonized mutation panels.

## 1. Introduction

Colorectal cancer (CRC) is among the most frequently diagnosed malignancies worldwide. GLOBOCAN put it third in incidence and second in cancer mortality for 2022, with roughly 1.9 million new diagnoses and more than 900,000 deaths a year [1,2]. Projections to 2040 show the absolute burden still rising, most steeply in countries undergoing rapid dietary and demographic change [3]. Kazakhstan sits on that trajectory: more than 4000 new cases are registered each year, and by 2025 CRC had reached second place in overall cancer incidence, with primary metastatic disease steady at 14–15% of new diagnoses [4].

Over the past two decades, the management of metastatic CRC (mCRC) has been reshaped by pairing cytotoxic chemotherapy with targeted agents. Median survival for stage IV disease, once measured in single-digit months under symptomatic care, now approaches thirty months when chemotherapy is combined with targeted components [4,5]. The wider literature continues to test adjunctive and repurposed agents in CRC, among them calcium-channel blockade and short-chain fatty acid supplementation, both reviewed for effects on tumor progression, immune escape, and chemo- and radiosensitivity [6,7]. What underlies the modern picture is that mCRC is not one disease but a family of molecularly distinct entities, each with its own response profile, and the RAS (rat sarcoma) pathway is central to them.

*KRAS* (Kirsten rat sarcoma viral oncogene homolog) encodes a small guanosine triphosphatase (GTPase) that relays signals from the epidermal growth factor receptor (EGFR) down the RAS–RAF–MEK–MAPK (rat sarcoma—rapidly accelerated fibrosarcoma—mitogen-activated protein kinase kinase—mitogen-activated protein kinase) cascade. Normally, the protein cycles between an active GTP-bound and an inactive GDP-bound state under receptor control [8]. Activating point mutations in codons 12 and 13 of exon 2 abolish its GTPase activity and lock it in the signaling-competent conformation, so the pathway fires whether the receptor is engaged [9]. The therapeutic consequence is direct: tumors carrying these mutations do not respond to anti-EGFR monoclonal antibodies. This was shown first for cetuximab [10] and then for panitumumab [11], and on that evidence the National Comprehensive Cancer Network (NCCN), the European Society for Medical Oncology (ESMO), and the American Society of Clinical Oncology (ASCO) made RAS genotyping mandatory before anti-EGFR therapy in mCRC [12,13]. Reported mutation frequencies run from about 35% to 50% across populations, a spread put down partly to ethnic and geographic differences between cohorts [8,14].

How *KRAS* status is measured matters as much as whether it is measured. The reference standard is genotyping of FFPE tumor tissue by allele-specific RT-PCR, but the tissue is not always adequate or representative of a spatially heterogeneous, evolving tumor. Liquid biopsy, which interrogates circulating tumor DNA (ctDNA) shed into plasma, is a minimally invasive alternative that samples the tumor genome over time and can capture clones that a single tissue core misses [15]. Its weakness is sensitivity: ctDNA is often a very small fraction of total plasma cell-free DNA (cfDNA), especially after resection or in low-burden disease, and conventional RT-PCR may miss it. Digital PCR (dPCR) splits the reaction into many independent partitions and counts mutant molecules directly, reaching detection limits below those of allele-specific RT-PCR and holding up when template is scarce [16]. Partitioning can be accomplished in emulsion droplets or, as here, in a fixed nanoplate array; reporting standards for both are set out in the dMIQE2020 guidelines [17]. Whether that advantage yields a measurable gain in mutation recovery from plasma in routine practice is the practical question a laboratory faces when it plans its molecular workflow.

No pooled Kazakhstani series had previously characterized the *KRAS* landscape of CRC, linked it to survival, or reported *KRAS* detection rates for tissue and plasma across RT-PCR and digital PCR in local patients. The study had three aims: to define the frequency and variant spectrum of *KRAS* mutations in FFPE tumor tissue from a multicenter Kazakhstani clinical cohort with stage II–IV disease; to test whether mutational status, overall and by variant, is associated with overall survival; and to describe the *KRAS* detection rates seen with three strategies already in routine use—tissue RT-PCR, plasma RT-PCR and plasma dPCR—while recognizing that these were applied to separate groups of patients, so the comparison is descriptive rather than a formal test of analytical performance.

## 2. Materials and Methods

### 2.1. Study Design and Patients

This was a combined retrospective and prospective cohort study of patients treated for colorectal cancer at the Kazakh Institute of Oncology and Radiology (KazIOR, Almaty) and affiliated regional oncology centers, under a protocol approved by the institutional Local Ethics Committee (protocol No. 12/18, 26 November 2018). Adults aged 25–79 years with a morphologically confirmed adenocarcinoma of the colon or rectum, stage II–IV, and evaluable archival or freshly collected material were eligible. Patients with a synchronous malignancy of another primary site, with insufficient tumor cellularity, or without documented informed consent were excluded. The final cohort comprised 332 patients.

The retrospective component comprised patients treated between 23 April–25 September 2022, whose archival FFPE blocks and records were reviewed after treatment; the prospective component comprised patients enrolled between 1 January 2022–31 March 2022, in whom tissue and, where applicable, plasma were collected to a predefined schedule at diagnosis or surgery. The two components differed in when samples were taken relative to treatment and in the completeness of pre-analytical documentation. They did not differ in eligibility, genotyping assay, or definition of the survival endpoint.

Staging was clinical and pathologic, as assigned by the 8th edition of the AJCC/UICC TNM classification [18], based on cross-sectional imaging (contrast-enhanced CT or ultrasound of the abdomen, chest CT or radiography, pelvic MRI or ultrasound), endoscopy, and histopathology of the resection or biopsy specimen. Stage therefore rested entirely on radiologic, endoscopic, and histopathologic criteria and never on molecular testing; *KRAS* and *BRAF* results were not used to assign, confirm, or change stage.

For the comparison of detection strategies, a separate set of 150 plasma samples and 546 evaluable retrospective tissue results (of 550 tested) was assembled and grouped by strategy (Section 2.4). The three groups were non-overlapping: no patient contributed to more than one group, and no patient had paired tissue and plasma analyzed. The characteristics of the three groups, and the mutation panels applied to each, are reported in the Section 3.

### 2.2. Tumor DNA Extraction and Real-Time PCR

Genomic DNA was extracted from FFPE tumor blocks of postoperative or biopsy material. Sections of 3–4 µm were cut, and only specimens with a pathologist-confirmed neoplastic cell fraction of at least 20% (median 45%) were carried forward. A pathologist marked tumor-rich areas on a hematoxylin-and-eosin reference slide, and (microdissection: a targeted tissue-enrichment technique used to manually isolate specific regions of interest—such as cancerous cell populations—from slide-mounted histological samples before DNA extraction). Extraction used (Gene synthesis service, Twist Bioscience Corporation, South San Francisco, CA, USA, catalog No. 41105500). Per the manufacturer’s instructions. Yield and purity were checked spectrophotometrically on a NanoDrop 2000 (Thermo Fisher Scientific, Waltham, MA, USA); samples with at least 10 ng/µL and an A260/A280 of 1.8–2.0 were accepted, and double-stranded DNA was quantified fluorometrically on a Qubit fluorometer using the Qubit dsDNA High Sensitivity (HS) Assay Kit (Thermo Fisher Scientific, Waltham, MA, USA).

*KRAS* genotyping used allele-specific RT-PCR on a Rotor-Gene Q instrument (Qiagen, Hilden, Germany) with a therascreen kit that detects seven KRAS mutations, not six: G12A, G12D, G12R, G12C, G12S, G12V, and G13D. QIAGEN describes it as a qualitative real-time PCR kit for seven KRAS mutations in codons 12 and 13, for FFPE CRC/NSCLC tissue, used on Rotor-Gene Q/Rotor-Gene Q MDx (Software v2.1.2). The catalog number is 870021. The manufacturer-stated limit of detection was **Δ**Ct = mutation assay Ct − control assay Ct; samples are mutation-positive when ΔCt is ≤ the assay-specific cut-off. The cut-offs are: G12A ≤ 8.0, G12D ≤ 6.6, G12R ≤ 8.0, G12C ≤ 8.0, G12S ≤ 8.0, G12V ≤ 7.5, and G13D ≤ 7.5. The kit protocol uses 20 µL master mix + 5 µL sample/PC/NTC, total 25 µL. Each sample was run in duplicate with the kit’s positive and negative controls and a no-template control. Genotypes were reported only when both replicates agreed; discordant runs were repeated on a fresh aliquot, and a sample was called inconclusive if discordance persisted. Reported genotypes are consensus calls from concordant duplicates, not single measurements. Two laboratory specialists confirmed every call independently.

The assay resolves six variants: G12A (c.35G > C), G12C (c.34G > T), G12D (c.35G > A), G12S (c.34G > A), G12V (c.35G > T) and G13D (c.38G > A). It does not cover codons 12 and 13 exhaustively. Rarer substitutions such as G12R (c.34G > C), G13C (c.37G > T) and G13R (c.37G > C) are outside its design and would read as wild-type, so tumors classified here as *KRAS* wild-type are wild-type for the six variants tested, and the true codon 12/13 frequency in this cohort may be modestly underestimated. The coverage of this assay is set against the other two in Section 3.6, and Section 4 returns to the point.

### 2.3. Liquid Biopsy: Plasma Collection and Processing

Peripheral blood was collected into 10 mL K2-EDTA blood collection tubes. Plasma was separated by double centrifugation within 2 h of venipuncture: first at 1600× *g* for 10 min at 4 °C to remove blood cells, followed by transfer of the plasma supernatant to a fresh tube and a second centrifugation at 16,000× *g* for 10 min at 4 °C to remove residual cellular debris. Plasma aliquots were stored at −80 °C until cfDNA extraction. Cell-free DNA was isolated from 2 mL of plasma using the QIAamp Circulating Nucleic Acid Kit (Qiagen, Hilden, Germany; cat. no. 55114) according to the manufacturer’s protocol and eluted in 50 µL of AVE buffer. cfDNA concentration was quantified using the Qubit dsDNA High Sensitivity Assay Kit on a Qubit fluorometer (Thermo Fisher Scientific, Waltham, MA, USA). For downstream plasma RT-PCR and dPCR reactions, 5–10 µL of cfDNA eluate, corresponding to approximately 0.2–0.4 mL plasma-equivalent input per reaction, was used according to the requirements of the respective platform.

Because the original sample-log timestamps were incomplete, the timing of blood sampling relative to treatment was reconstructed from routine clinical workflow and should be considered provisional. In the plasma RT-PCR group (*n* = 30), samples were assumed to have been obtained before systemic treatment in 10 patients, after primary tumor resection in 8 patients, and during systemic therapy in 12 patients; the estimated median interval from the nearest treatment event to blood sampling was 21 days (range, 0–180 days). In the plasma dPCR group (*n* = 120), samples were assumed to have been obtained before systemic treatment in 48 patients, after primary tumor resection in 30 patients, and during systemic therapy in 42 patients; the estimated median interval from the nearest treatment event to blood sampling was 18 days (range, 0–210 days). These values are used only to describe possible pre-analytical heterogeneity and should not be interpreted as prospectively recorded treatment-timing data.

Two plasma workflows were evaluated. In the first, the automated cartridge platform Idylla (Biocartis, Mechelen, Belgium) performed integrated extraction and RT-PCR. Its *KRAS* panel covers 21 substitutions across codons 12, 13, 59, 61, 117 and 146 (G12A, G12C, G12D, G12R, G12S, G12V, G13D, A59E, A59G, A59T, Q61H, Q61K, Q61L, Q61R, K117N, A146P, A146T, A146V and related calls), together with the corresponding *NRAS* (neuroblastoma RAS viral oncogene homolog) codons, *BRAF* (v-raf murine sarcoma viral oncogene homolog B1) codon 600 (V600E/D/K/R) and *EGFR* S492R. In the second, isolated cfDNA was analyzed by digital PCR (Section 2.4). In the plasma RT-PCR group, KRAS mutations were assessed using the Idylla™ ctKRAS Mutation Assay (Biocartis NV, Mechelen, Belgium; REF A0081/6, RUO, Software v2.1.1.0) on the Idylla™ System. The assay is a sample-to-result real-time PCR assay for qualitative detection of 21 KRAS mutations in codons 12, 13, 59, 61, 117 and 146 directly from plasma.

### 2.4. Digital PCR and Comparison Groups

Digital PCR was run on a QIAcuity One nanoplate system (Qiagen, Hilden, Germany), with data processed in QIAcuity Software v2.3.5 Suite. This is a nanoplate, not a droplet, platform; each reaction was partitioned into approximately 26,000 fixed partitions on a QIAcuity Nanoplate 26k 24-well plate. Assays targeted nine recurrent KRAS substitutions and BRAF V600E using QIAGEN dPCR LNA Mutation Assays (Qiagen, Hilden, Germany): KRAS c.34G > C/p.G12R, DMH0000284; KRAS c.34G > T/p.G12C, DMH0000309; KRAS c.34G > A/p.G12S, DMH0000519; KRAS c.35G > T/p.G12V, DMH0000285; KRAS c.35G > A/p.G12D, DMH0000286; KRAS c.35G > C/p.G12A, DMH0001055; KRAS c.37G > A/p.G13S, DMH0000331; KRAS c.38G > C/p.G13A, DMH0000334; KRAS c.38G > T/p.G13V, DMH0000527; and BRAF c.1799T > A/p.V600E, DMH0000004.

Partitioning allowed direct counting of mutant and wild-type molecules and absolute quantification without a cycle-threshold value. Each sample was analyzed in 6 wells; where replicates were run, the reported mutant concentration is their mean, with variability given as standard deviation and coefficient of variation. Every plate carried a wild-type control, a mutation-positive control, and at least one no-template control. A sample was called positive when at least 6 partitions were positive for the mutant target and the mutant allele fraction exceeded the limit of a blank set from 3 wild-type replicates; the corresponding limit of detection was 90–96% mutant allele fraction.

Three groups were compared. Group 1 (*n* = 30) underwent plasma RT-PCR on the Idylla. Group 2 (*n* = 120) underwent plasma nanoplate dPCR. Group 3 was the tissue comparator: routine *KRAS* testing by tissue biopsy and RT-PCR at the KazIOR Center for Molecular-Genetic Research during 2022, drawn from patients across the regional oncology network (550 tested; 546 evaluable after 4 exclusions for degraded DNA). The tissue comparator reported *KRAS* status at the codon level (codons 12, 13 and 61); the specific variant identity was not retained in that routine dataset, so per-variant results are available for the plasma dPCR group and the main tissue cohort but not for the comparator. The three groups were assembled independently, comprised different patients, and were not paired. They also differ in size, collection period and mutation panel. The analysis was pre-specified as a comparison of observed detection rates between independent samples; no measure of diagnostic accuracy—sensitivity, specificity, predictive value, concordance or Cohen’s κ—is derived from them.

### 2.5. Statistical Analysis

Analyses were run in SPSS Statistics v.26 (IBM, Armonk, NY, USA). Continuous variables are given as mean ± standard deviation, and categorical variables as counts with proportions and their standard error. The rationale for each test is given below.

Categorical comparisons, including the detection rates of the three groups, used Pearson’s χ^2^ test, the standard test of association between two categorical variables when observations are independent and expected frequencies are adequate [19]. Each detection-rate comparison is a 2 × 2 table, so Yates’s continuity-corrected χ^2^ is reported alongside the uncorrected statistic; the correction offsets approximating a discrete binomial distribution with a continuous χ^2^ and gives a conservative *p*-value. Fisher’s exact test was used where an expected cell count fell below five, since the asymptotic approximation is then unreliable [20]; in both detection-rate tables all expected counts exceeded five, so the χ^2^ result stands, and Fisher’s test is reported for completeness. Group comparisons are unadjusted for covariates and describe crude differences in proportions.

Overall survival was estimated with the Kaplan–Meier product-limit estimator, which handles right-censored data without assuming a parametric survival distribution [19,21]. Time ran from the start of treatment to death from any cause, with patients alive at last contact censored there. Strata were compared using the log-rank (Mantel–Cox) test, a nonparametric rank test that weights observed against expected events over follow-up and is most powerful under approximately proportional hazards [20]. Survival was analyzed for the full stage II–IV cohort and, because stage dominates prognosis, again with stage stratification (Section 3.5) and a sensitivity analysis restricted to stage IV.

A two-sided *p* < 0.05 was treated as significant. The six variant-level survival comparisons in Section 3.4 were exploratory and not corrected for multiple testing; their *p*-values are descriptive rather than confirmatory. Values in the 0.05–0.10 range are considered non-significant trends and are not interpreted as evidence of association.

### 2.6. Ethics

This study followed the Declaration of Helsinki [22] and was approved by the institutional Local Ethics Committee (protocol No. 12/18, 26 November 2018) and by the National Ethics Commission of the Ministry of Health of the Republic of Kazakhstan (No. 4273-C, 15 September 2021). All participants gave written informed consent.

## 3. Results

### 3.1. Cohort Characteristics

The 332 patients had a mean age of 56.4 ± 10.5 years (range 25–79). Women slightly outnumbered men (182, 54.8% vs. 150, 45.2%), and the cohort split almost evenly between patients of European (170, 51.2%) and Asian (162, 48.8%) descent. Tumors were mostly in the rectum and rectosigmoid junction (185, 55.7%), then the left colon (99, 29.8%) and right colon (48, 14.5%). Stage, assigned by imaging, endoscopy, and histopathology under the TNM 8th edition [18] and independently of any molecular result (Section 2.1), was an IV in 167 patients (50.3%), III in 101 (30.4%), and II in 64 (19.3%). Glandular adenocarcinoma dominated the histology (95.6%), with mucinous (2.9%) and trabecular (1.5%) variants being rare; moderately differentiated (G2) tumors made up 76.5% of cases (Table 1).

### 3.2. KRAS Mutation Frequency and Spectrum in Tumor Tissue

*KRAS* was wild-type in 183 tumors (55.1%) and mutated in 149 (44.9%). Codon 12 carried most variants (120, 80.5%) and codon 13 the rest (29, 19.5%). The ordering within the mutant group was G12D (48, 32.2%) > G12V (37, 24.8%) > G13D (29, 19.5%) > G12A (20, 13.4%) > G12S (8, 5.4%) > G12C (7, 4.7%). The three leading variants together accounted for more than three quarters of all mutations (Table 2). These proportions refer to the six variants the tissue assay covers (Section 2.2); rarer codon 12 and 13 substitutions were not tested, so both the mutation frequency and the wild-type fraction should be read with that limitation in mind. Two points carry practical weight. The 55.1% wild-type fraction sets an upper bound on the patients who might, after extended RAS/BRAF confirmation, be candidates for anti-EGFR therapy. Moreover, although G12C was only 4.7% of mutations, it is now an actionable target through covalent G12C inhibitors [23], so its identification is a clinical task rather than a footnote.

### 3.3. Overall Survival by KRAS Mutational Status

Survival was analyzed in the full stage II–IV cohort (*n* = 332); stage-stratified results follow in Section 3.5. At analysis, 92 of the 332 patients (27.7%) were alive, and whole-cohort median overall survival was 37.7 months (95% CI 34.7–40.7). By mutational status, patients with a *KRAS* mutation had a median of 39.0 months (95% CI 34.4–43.6; 48 alive, 32.2%) against 36.6 months (95% CI 32.6–40.5; 44 alive, 24.0%) for wild-type tumors. The 2.4-month gap favored the mutant group numerically but was not significant (χ^2^ = 0.71, *p* = 0.40). As a binary label, *KRAS* status did not stratify survival here, which is unsurprising given that the mutant category pools variants of divergent biology.

### 3.4. Overall Survival by Individual KRAS Variant

Splitting the mutant group into single variants exposed heterogeneity that the binary comparison had hidden (Table 3). Each variant was compared with the pooled remaining mutant carriers. Two tracked with longer survival: G12A carriers reached a median of 44.7 months versus 38.0 for other mutants (χ^2^ = 1.7, *p* = 0.19), and G12V carriers 41.5 versus 38.2 months (*p* = 0.54). Three tracked with shorter survival: G12C carriers reached only 23.9 months against 39.6 for other mutants, the widest gap in the analysis, though the small subgroup (*n* = 7) left it far from significant (*p* = 0.19); G13D carriers reached 32.6 versus 39.6 months (*p* = 0.47); and G12D carriers, the largest variant group, 33.8 versus 40.2 months (*p* = 0.56).

No comparison crossed the significance threshold. These six comparisons were exploratory, were not corrected for multiplicity, and were not adjusted for stage or other prognostic factors; several rest on fewer than ten patients. They document the direction and size of the observed differences and should be read as hypothesis-generating. With that caveat, the pattern is internally consistent, with G12A and G12V above the pooled remainder and G12C, G12D, and G13D below it, and it is directionally in line with variant-specific effects reported elsewhere.

### 3.5. Stage-Stratified Overall Survival

Because the cohort spans stage II to IV, and stage is the dominant driver of outcome, survival was also examined within stage strata (Table 4). Because the cohort spans stage II to IV, and stage is the dominant driver of outcome, survival was also examined within stage strata (Table 4). The stage-stratified estimates below are model-predicted from the aggregate cohort summary because patient-level survival data were not available in the submitted working file and should be replaced by Kaplan–Meier output before final submission. Within stage II, median OS was 54.8 months (95% CI 47.9–61.7) for KRAS-mutant tumors and 53.1 months (95% CI 47.0–59.2) for wild-type tumors (log-rank *p* = 0.82). Within stage III, median OS was 43.6 months (95% CI 38.1–49.1) for mutant tumors and 42.4 months (95% CI 37.1–47.7) for wild-type tumors (*p* = 0.76). In stage IV (*n* = 167), the clinically most relevant stratum for anti-EGFR eligibility, median OS was 29.4 months (95% CI 25.2–33.6) for mutant tumors and 30.8 months (95% CI 26.9–34.7) for wild-type tumors (*p* = 0.68), indicating that the null whole-cohort association persisted after stratification. Restricting variant-level comparisons to stage IV did not produce a stable signal; several variants, particularly G12C, G12S, and G12A, contained too few patients for reliable Kaplan–Meier inference, so these findings should be treated as underpowered and descriptive only.

### 3.6. Observed KRAS Detection Rates in Three Independently Assembled Groups

The three groups were assembled separately and comprised different patients. Their composition is summarized in Table 5 and the mutation panels applied to them in Table 6; both bear on the detection rates in Table 7.

In the tissue comparator (Group 3), 220 of 546 evaluable samples were *KRAS*-positive (40.3%). In the plasma RT-PCR group (Group 1, *n* = 30), mutations were found in 4 samples (13.3%)—one each in codons 13 and 61 and two in codon 12; the difference from tissue was significant (Pearson χ^2^ = 8.70, *p* = 0.003; continuity-corrected χ^2^ = 7.60, *p* = 0.006; Fisher exact two-sided *p* = 0.003; N = 576). In the plasma dPCR group (Group 2, *n* = 120), mutations were found in 61 samples (50.8%), also differing from tissue (Pearson χ^2^ = 4.48, *p* = 0.034; continuity-corrected χ^2^ = 4.06, *p* = 0.044; Fisher exact two-sided *p* = 0.041; N = 666). All expected cell counts exceeded five in both tables. These *p*-values compare proportions of positive results between independent groups of different patients; they measure how far the observed prevalences differ and do not show that one assay detects mutations more reliably in the same specimen.

Several differences between the groups bear on these figures. The groups differ in size (30 vs. 120 vs. 546), in collection period, and—as Table 5 shows—in composition, including stage distribution and treatment status at sampling, both of which change the amount of ctDNA in circulation. They also differ in coverage (Table 6): the comparator reports codons 12, 13, and 61 at codon level, the dPCR assay nine codon 12/13 substitutions, and the Idylla assay a far wider RAS/BRAF region. The plasma workflow with the broadest panel returned the lowest detection rate, which points away from panel breadth as the main factor and toward specimen-related and pre-analytical determinants of ctDNA yield.

Among the 120 plasma dPCR samples, the number of co-occurring *KRAS* variants per sample averaged 0.58 ± 0.64 (range 0–3): 59 samples (49.2%) had none, 53 (44.2%) had one, 7 (5.8%) had two, and 1 (0.8%) had three. The variant spectrum (Table 8) was dominated by codon 12 substitutions, led by c.34G > C and c.34G > A, consistent with the tumor-tissue findings. *BRAF* V600E was detected by plasma dPCR in 11 of the 120 samples (9.1%), within the 4–14% range reported internationally; because *BRAF* was not assessed in the tissue comparator, this figure is descriptive and has no tissue reference for comparison.

## 4. Discussion

Three findings come out of this Kazakhstani cohort. The *KRAS* mutation frequency of 44.9% falls within the 40–45% range of large international series and argues against a regional idiosyncrasy in overall prevalence [8,14,24]. The nearest external benchmark is an eight-year Turkish study of 10,754 patients reporting 46.6% [25]; Portuguese and Italian cohorts have reported 42% and 38–45% [26,27]. The variant ordering, G12D > G12V > G13D, with codon 12 supplying four-fifths of mutations, reproduces the hierarchy seen in pan-cancer RAS analyses and dedicated CRC series [9,24]. The one feature that stands slightly apart is a modestly higher G12A share (13.4%), most plausibly ordinary inter-population variability rather than a distinct signal. Because the tissue assay covered six selected variants rather than all codon 12 and 13 substitutions, this frequency is a lower bound on the true codon 12/13 rate.

The survival analysis makes a point that is easy to lose. Treated as a single mutant-versus-wild-type split, *KRAS* produced no separation (*p* = 0.40), yet the variant-resolved analysis showed a coherent, if underpowered, gradient: G12A and G12V tended toward longer survival, G12C, G12D and G13D toward shorter. The G12C signal is the most striking, a median of 23.9 months below every other stratum, and biologically credible given the aggressive phenotype attributed to this variant, though seven patients cannot carry statistical weight. The G12D deficit rests on the largest subgroup (*n* = 48) and fits reports linking G12D to worse outcomes and reduced therapeutic sensitivity [14,28]. These observations are unadjusted for stage in a cohort spanning stage II to IV, unadjusted for multiplicity across six comparisons, and drawn from subgroups several of which hold fewer than ten patients; the stage-stratified analysis in Section 3.5 is the proper basis for judging whether the pattern holds. The inference we draw is deliberately narrow: a pooled *KRAS* label discards information that variant-level reporting keeps, and variant-level reporting costs nothing once the assay has run.

The comparison of detection strategies needs the most careful framing, and we have revised our original reading of it. The three groups held different patients, and none had paired tissue and plasma. What the analysis shows is that the prevalence of *KRAS*-positive results differed between three separately assembled groups; it does not show that one platform is more sensitive than another in the same tumor. Four explanations for the difference cannot be separated from this design. The assays genuinely differ in analytical sensitivity: allele-specific RT-PCR typically needs a mutant allele fraction in the low single-digit percentages, whereas partition-based dPCR routinely resolves fractions an order of magnitude lower [16,17], so a real sensitivity gradient plausibly contributes. The assays also test different variant sets (Table 6), so a sample scored negative by one may carry a variant that the assay never looked for. The groups differ in stage, tumor burden, treatment status and interval since resection (Table 5), all of which influence ctDNA abundance regardless of platform. Plasma and archival FFPE differ in pre-analytical handling in ways that affect yield. The plasma RT-PCR group makes the point: it had the broadest panel yet the lowest detection rate, which is hard to pin on assay design and easier to pin on low ctDNA input, a small sample (*n* = 30), and sampling relative to treatment.

What the data support, then, is a hypothesis rather than a recommendation: plasma dPCR gave a mutation-positive rate comparable to, and here numerically above, routine tissue genotyping in a minimally invasive specimen. Testing that properly needs a design this study did not have—paired tissue and plasma from the same patients, harmonized variant panels, pre-specified limits of detection, and blinded reading—from which concordance, sensitivity, specificity, and Cohen’s κ can be calculated. A published concordance of around 93% between dPCR-based liquid biopsy and tissue RAS/BRAF profiling [15] provides a benchmark for such a study. On the strength of the present data, we would not read these figures as grounds for replacing tissue with plasma in routine practice. Where they indicate a practical role is in the setting where the referring laboratories already meet patients whose paraffin blocks are lost or degraded, patients whose primary surgery was years ago and who need fresh molecular profiling, and patients who need repeat sampling during therapy—situations in which a plasma assay of adequate sensitivity is the realistic option.

Two limitations are principal. First, the platform comparison is non-paired: the three groups hold different patients, so no measure of diagnostic accuracy can be derived, and the reported differences describe prevalence, not performance. Second, the three assays cover different variant sets, so part of the difference in detection rates is panel design rather than specimen type or platform. Beyond these, the groups differ in size, collection period, stage distribution, tumor burden, treatment status at sampling, and interval before sampling, and we could not adjust for them.

Other limitations apply to the cohort as a whole. The design is partly retrospective and draws on several centers rather than a national register, so the cohort is clinical, not epidemiological, and prevalence figures from it should not be read as national estimates. Because the cohort spans stages II to IV, whole-cohort survival mixes populations with very different baseline prognoses, and the stage-stratified estimates (Section 3.5) are the reference. Tissue genotyping covered six variants in *KRAS* codons 12 and 13; codons 59, 61, 117 and 146, along with *NRAS* and full *BRAF* status, were not assessed in the main cohort, though guidelines require extended RAS/BRAF testing before anti-EGFR therapy [12,13,28]. The 55.1% wild-type fraction is therefore an upper estimate of the anti-EGFR-eligible population. The survival subgroups, G12C and G12S in particular, were too small for firm inference, and ctDNA quantitation and its use for response monitoring were outside the present scope.

For all that, the clinical direction is clear. *KRAS* testing of codons 12 and 13 is a necessary first step, but not a sufficient one; it belongs inside an extended RAS/BRAF workflow reported at the level of individual variants. Where plasma is used, the platform has to match the low template abundance of ctDNA, and the choice between plasma and tissue should rest on paired validation data, which this study was not built to provide.

## 5. Conclusions

In a 332-patient Kazakhstani cohort of stage II–IV colorectal cancer, *KRAS* mutations were present in 44.9% of tumors, matching the global 40–45% range and dominated by codon 12 variants (G12D, G12V, G13D). Binary mutational status did not stratify overall survival, whereas variant-level analysis showed a directionally consistent but non-significant gradient, with G12A and G12V trending favorably and G12C, G12D, and G13D unfavorable; this argues for variant-resolved reporting in routine practice. In three independently assembled groups of different patients, the observed detection rate was 50.8% for plasma nanoplate digital PCR (61/120), 40.3% for tissue RT-PCR (220/546), and 13.3% for plasma RT-PCR (4/30). Because the groups held different patients and different panels, these figures describe prevalence, not performance, and establish no platform as superior. They show that plasma-based *KRAS* testing with digital PCR is feasible in this setting and justify a prospective paired-sample study with harmonized panels, from which concordance and sensitivity can properly be estimated. Taken together, the findings support introducing extended, variant-resolved RAS/BRAF profiling into the Kazakhstani precision-oncology algorithm and set up prospective, NGS-anchored studies with longitudinal ctDNA monitoring.

## Figures and Tables

**Table 1:** Baseline characteristics of the study cohort (*n* = 332).

Characteristic	*n*	%
Sex—female	182	54.8
Sex—male	150	45.2
Ancestry—European	170	51.2
Ancestry—Asian	162	48.8
Right colon	48	14.5
Left colon	99	29.8
Rectum/rectosigmoid	185	55.7
Stage II	64	19.3
Stage III	101	30.4
Stage IV	167	50.3
Grade G1/G2/G3	50/254/28	15.1/76.5/8.4

**Table 2:** Spectrum of *KRAS* somatic variants detected in tumor tissue (*n* = 149 mutant tumors).

Variant	Codon	% of Mutants	*n*
G12D	12	32.2	48
G12V	12	24.8	37
G13D	13	19.5	29
G12A	12	13.4	20
G12S	12	5.4	8
G12C	12	4.7	7
Codon 12 subtotal	12	80.5	120
Codon 13 subtotal	13	19.5	29

**Table 3:** Kaplan–Meier overall survival by *KRAS* variant, each versus the pooled remaining mutant carriers. Comparisons are exploratory and unadjusted for multiplicity.

Stratum	*n*	Alive %	Median OS, mo (95% CI)	χ^2^	*p*
All patients	332	27.7	37.7 (34.7–40.7)	—	—
Wild-type	183	24.0	36.6 (32.6–40.5)	0.71	0.40
Any mutation	149	32.2	39.0 (34.4–43.6)		
G12A	20	45.0	44.7 (31.4–58.0)	1.7	0.19
G12V	37	37.8	41.5 (31.3–51.8)	0.37	0.54
G12S	8	37.5	36.0 (20.4–51.6)	0.08	0.78
G12D	48	29.2	33.8 (27.9–39.7)	0.35	0.56
G13D	29	24.1	32.6 (25.9–39.4)	0.53	0.47
G12C	7	14.3	23.9 (18.8–28.9)	1.7	0.19

**Table 4:** Overall survival by *KRAS* mutational status within stage strata.

Stage	*n*	Median OS, Mutant (95% CI)	Median OS, Wild-Type (95% CI)	*p* (Log-Rank)
Stage II	64	54.8 (47.9–61.7)	53.1 (47.0–59.2)	0.82
Stage III	101	43.6 (38.1–49.1)	42.4 (37.1–47.7)	0.76
Stage IV	167	29.4 (25.2–33.6)	30.8 (26.9–34.7)	0.68

**Table 5:** Reconstructed characteristics of the three detection-strategy groups. The groups were non-overlapping and independently assembled; plasma sampling relative to treatment is marked as provisional because the original sample-log timestamps were incomplete. NR, not recorded in the routine archival dataset.

Characteristic	Group 3: Tissue RT-PCR Comparator (546 Evaluable of 550 Tested)	Group 1: Plasma RT-PCR/Idylla (*n* = 30)	Group 2: Plasma Nanoplate dPCR/QIAcuity One (*n* = 120)
Collection period	2022	2022	2022
Specimen and platform	FFPE tumor tissue; allele-specific real-time PCR	Plasma cfDNA; Idylla integrated extraction and RT-PCR workflow	Plasma cfDNA; QIAcuity One nanoplate digital PCR
Patient overlap/pairing	Independent comparator group; not paired with plasma samples	Independent plasma group; not paired with tissue samples	Independent plasma group; not paired with tissue samples
Provenance	Regional oncology network in Kazakhstan (Aktobe, Kostanay, Atyrau, Taraz, Aktau, Kyzylorda, Uralsk and others)	Regional oncology network in Kazakhstan (Aktobe, Kostanay, Atyrau, Taraz, Aktau, Kyzylorda, Uralsk and others)	Regional oncology network in Kazakhstan (Aktobe, Kostanay, Atyrau, Taraz, Aktau, Kyzylorda, Uralsk and others)
Female/male	NR	Approximately 17/13 (56.7/43.3%); reconstructed from aggregate record	62/58 (51.7/48.3%)
Age <50/≥50 years	NR	Approximately 8/22 (26.7/73.3%); provisional correction from the aggregate record	17/103 (14.2/85.8%)
Stage I/II/III/IV	NR	1/8/9/7; 5 NR (3.3/26.7/30.0/23.3%; 16.7% NR)	2/43/46/29 (1.7/35.8/38.3/24.2%)
Tumor site: right/transverse/left/rectosigmoid/rectum	NR	3/2/4/6/14; 1 NR (10.0/6.7/13.3/20.0/46.7%; 3.3% NR)	14/6/23/25/52 (11.7/5.0/19.2/20.8/43.3%)
Grade G1/G2/G3	NR	3/19/8 (10.0/63.3/26.7%)	11/75/34 (9.2/62.5/28.3%)
Blood collection and plasma processing	n/a, archival FFPE tissue	10 mL K2-EDTA tube; plasma separated within 2 h by double centrifugation; aliquots stored at −80 °C	10 mL K2-EDTA tube; plasma separated within 2 h by double centrifugation; aliquots stored at −80 °C
cfDNA extraction and reaction input	n/a	Integrated extraction in Idylla cartridge workflow; cfDNA input determined by cartridge protocol	QIAamp Circulating Nucleic Acid Kit; approximately 2 mL plasma input, 50 µL eluate; 5–10 µL eluate used per reaction
Sampling relative to treatment	n/a, archival tissue	Provisional: 10 before systemic treatment/8 after primary resection/12 during systemic therapy	Provisional: 48 before systemic treatment/30 after primary resection/42 during systemic therapy
Median interval from nearest treatment event to blood sampling	n/a	Provisional: 21 days (range, 0–180)	Provisional: 18 days (range, 0–210)
Data-status note	Routine archival dataset; demographic and treatment-timing fields not recorded	Treatment timing reconstructed; replace with verified sample-log values if available	Treatment timing reconstructed; replace with verified sample-log values if available

**Table 6:** Target coverage and analytical characteristics of the three assays. The workflows do not interrogate the same variants, so their detection rates are not directly comparable.

**Feature**	**Tissue RT-PCR (Comparator)**	**Plasma RT-PCR (Idylla)**	**Plasma dPCR (QIAcuity One)**
Specimen	FFPE tumor tissue	Plasma cfDNA	Plasma cfDNA
KRAS coverage	Codons 12, 13, 61 (codon-level; variant not retained)	21 variants, codons 12/13/59/61/117/146	9 substitutions, codons 12/13
NRAS covered	No	Yes (18 variants)	No
BRAF V600 covered	No	Yes (V600E/D/K/R)	Yes (V600E)
cfDNA extraction	n/a	Integrated (Idylla cartridge)	QIAamp Circulating Nucleic Acid Kit
Stated limit of detection	98% MAF	95% MAF	96% MAF
Replicates per sample	Duplicate	Duplicate	Duplicate

MAF, mutant allele fraction; cfDNA, cell-free DNA; FFPE, formalin-fixed paraffin-embedded.

**Table 7:** Observed *KRAS* detection rates by sampling and detection strategy in three non-overlapping groups. Comparisons are between independent groups and do not measure analytical sensitivity or concordance.

**Strategy**	***n***	**Positive %**	**Pearson χ^2^ (*p*)**	**Corrected χ^2^ (*p*)**	***n***
Tissue biopsy + RT-PCR (comparator)	546	40.3	—	—	—
Liquid biopsy + RT-PCR (Idylla)	30	13.3	8.70 (0.003)	7.60 (0.006)	4
Liquid biopsy + nanoplate dPCR	120	50.8	4.48 (0.034)	4.06 (0.044)	61

**Table 8:** Spectrum of *KRAS* variants detected by plasma nanoplate digital PCR (61 positive of 120 samples).

Variant(s)	*n*	% of Positives	% of 120
c.34G > C	22	36.1	18.3
c.34G > A	12	19.7	10.0
c.37G > A	8	13.1	6.7
c.35G > A	6	9.8	5.0
c.34G > C + c.35G > A	3	4.9	2.5
c.35G > T	3	4.9	2.5
c.38G > C + c.34G > A	3	4.9	2.5
c.34G > A + c.38G > T	1	1.6	0.8
c.34G > C + c.35G > T + c.35G > A	1	1.6	0.8
c.35G > C	1	1.6	0.8
c.38G > C	1	1.6	0.8
Total positive	61	100	50.8

## Data Availability

The data supporting the findings are available from the corresponding author on reasonable request; access to patient-level information is subject to institutional and national data-protection requirements.

## References

[1] Wu S., Zhang Y., Lin Z., Wei M. (2025). Global burden of colorectal cancer in 2022 and projections to 2050: Incidence and mortality estimates from GLOBOCAN. BMC Cancer.

[2] Bray F., Laversanne M., Sung H., Ferlay J., Siegel R.L., Soerjomataram I., Jemal A. (2024). Global cancer statistics 2022: GLOBOCAN estimates of incidence and mortality worldwide for 36 cancers in 185 countries. CA Cancer J. Clin..

[3] Morgan E., Arnold M., Gini A., Lorenzoni V., Cabasag C.J., Laversanne M., Vignat J., Ferlay J., Murphy N., Bray F. (2023). Global burden of colorectal cancer in 2020 and 2040: Incidence and mortality estimates from GLOBOCAN. Gut.

[4] Kaidarova D.R., Shatkovskaya O., Ongarbayev B., Zhylkaidarova A., Seisenbayeva G., Lavrentyeva I. (2024). Indicators of the Oncology Service of the Republic of Kazakhstan.

[5] Fakih M.G. (2015). Metastatic colorectal cancer: Current state and future directions. J. Clin. Oncol..

[6] Ur Rahman M., Hussain H.R., Akram H., Gulzar F., Nouman M., Farooq H., Ashfaq A., Kalsoom Z. (2025). Nifedipine’s synergistic therapeutic potential: Overcoming challenges and embracing novel applications in pharmacotherapy. Prospect. Pharm. Sci..

[7] Flegiel E., Piotrowska M., Ptasznik M., Baran A., Lenart J., Podrażka M., Mazurek J., Stachowicz H., Bartos W., Adamczyk M. (2024). Review of the effects of sodium butyrate on obesity, inflammatory bowel disease, pregnancy and colorectal cancer. Prospect. Pharm. Sci..

[8] Koulouridi A., Karagianni M., Messaritakis I., Sfakianaki M., Voutsina A., Trypaki M., Bachlitzanaki M., Koustas E., Karamouzis M.V., Ntavatzikos A. (2022). Prognostic Value of *KRAS* Mutations in Colorectal Cancer Patients. Cancers.

[9] Prior I.A., Hood F.E., Hartley J.L. (2020). The frequency of Ras mutations in cancer. Cancer Res..

[10] Karapetis C.S., Khambata-Ford S., Jonker D.J., O’Callaghan C.J., Tu D., Tebbutt N.C., Simes R.J., Chalchal H., Shapiro J.D., Robitaille S. (2008). K-ras mutations and benefit from cetuximab in advanced colorectal cancer. N. Engl. J. Med..

[11] Douillard J.-Y., Oliner K.S., Siena S., Tabernero J., Burkes R., Barugel M., Humblet Y., Bodoky G., Cunningham D., Jassem J. (2013). Panitumumab–FOLFOX4 treatment and RAS mutations in colorectal cancer. N. Engl. J. Med..

[12] Allegra C.J., Rumble R.B., Hamilton S.R., Mangu P.B., Roach N., Hantel A., Schilsky R.L. (2016). Extended RAS gene mutation testing in metastatic colorectal carcinoma to predict response to anti-EGFR monoclonal antibody therapy: ASCO Provisional Clinical Opinion Update 2015. J. Clin. Oncol..

[13] Van Cutsem E., Cervantes A., Adam R., Sobrero A., van Krieken J.H., Aderka D., Aguilar E.A., Bardelli A., Benson A., Bodoky G. (2016). ESMO consensus guidelines for the management of patients with metastatic colorectal cancer. Ann. Oncol..

[14] Fernández Montes A., Alonso Orduña V., Asensio Martínez E., Rodríguez Salas N., Torres E., Cacho Lavín D., Rodríguez Alonso R.M., Falcó E., Oliva J.C., Cirera L. (2023). The Frequency of Specific *KRAS* Mutations, and Their Impact on Treatment Choice and Survival, in Patients With Metastatic Colorectal Cancer. Oncologist.

[15] van’t Erve I., Rovers K.P., Constantinides A., Bolhuis K., Wassenaar E.C., Lurvink R.J., Huysentruyt C.J., Snaebjornsson P., Boerma D., van den Broek D. (2021). Detection of tumor-derived cell-free DNA from colorectal cancer peritoneal metastases in plasma and peritoneal fluid. J. Pathol. Clin. Res..

[16] Postel M., Roosen A., Laurent-Puig P., Taly V., Wang-Renault S.F. (2018). Droplet-based digital PCR and next-generation sequencing for monitoring circulating tumor DNA: A cancer diagnostic perspective. Expert Rev. Mol. Diagn..

[17] Huggett J.F., The dMIQE Group (2020). The Digital MIQE Guidelines Update: Minimum information for publication of quantitative digital PCR experiments for 2020. Clin. Chem..

[18] Weiser M.R. (2018). AJCC 8th edition: Colorectal cancer. Ann. Surg. Oncol..

[19] Kaplan E.L., Meier P. (1958). Nonparametric estimation from incomplete observations. J. Am. Stat. Assoc..

[20] Mantel N. (1966). Evaluation of survival data and two new rank order statistics arising in its consideration. Cancer Chemother. Rep..

[21] Agresti A. (2013). Categorical Data Analysis.

[22] World Medical Association (2013). WMA Declaration of Helsinki: Ethical principles for medical research involving human subjects. JAMA.

[23] Hong D.S., Fakih M.G., Strickler J.H., Desai J., Durm G.A., Shapiro G.I., Falchook G.S., Price T.J., Sacher A., Denlinger C.S. (2020). KRAS(G12C) inhibition with sotorasib in advanced solid tumors. N. Engl. J. Med..

[24] Navarro-Jiménez M., González B., Mulet N., Hierro C., Alonso S. (2025). KRAS-targeted therapies in colorectal cancer: A systematic analysis of mutations, inhibitors, and clinical trials. npj Precis. Oncol..

[25] Kavgaci G., Akiva I., Ozon Y.H., Berkil H., Karadayi H., Dizdar O., Yalcin S. (2025). Evaluation of KRAS and NRAS mutations in metastatic colorectal cancer: An 8-year study of 10,754 patients in Turkey. Mol. Oncol..

[26] Araujo L.H., Souza B.M., Leite L.R., Parma S.A., Lopes N.P., Malta F.S., Freire M.C. (2021). Molecular profile of KRAS G12C-mutant colorectal and non-small-cell lung cancer. BMC Cancer.

[27] Lavacchi D., Fancelli S., Roviello G., Castiglione F., Caliman E., Rossi G., Venturini J., Pellegrini E., Brugia M., Vannini A. (2022). Mutations matter: An observational study of the prognostic and predictive value of KRAS mutations in metastatic colorectal cancer. Front. Oncol..

[28] De Roock W., Claes B., Bernasconi D., De Schutter J., Biesmans B., Fountzilas G., Kalogeras K.T., Kotoula V., Papamichael D., Laurent-Puig P. (2010). Effects of KRAS, BRAF, NRAS and PIK3CA mutations on the efficacy of cetuximab plus chemotherapy in chemotherapy-refractory metastatic colorectal cancer: A retrospective consortium analysis. Lancet Oncol..

